# Assessment of the Impact of Cardiac Implantable Electronic Devices on Patients' Quality of Life

**DOI:** 10.7759/cureus.57261

**Published:** 2024-03-30

**Authors:** Nika Kuridze, Mikheil Tsverava, Tengiz Verulava

**Affiliations:** 1 Faculty of Clinical and Translational Medicine, Ivane Javakhishvili Tbilisi State University, Tbilisi, GEO; 2 Department of Rhythmology, Acad. G. Chapidze Emergency Cardiology Center, Tbilisi, GEO; 3 Faculty of Internal Medicine, Ivane Javakhishvili Tbilisi State University, Tbilisi, GEO; 4 Department of Internal Medicine, Acad. G. Chapidze Emergency Cardiology Center, Tbilisi, GEO; 5 School of Medicine, Caucasus University, Tbilisi, GEO

**Keywords:** cardiac resynchronization therapy (crt), implantable cardioverter-defibrillator, cardiac pacemaker, cardiac implantable electronic device (cied), quality of life (qol)

## Abstract

Introduction: Assessing the quality of life serves as a crucial metric during various therapeutic or surgical procedures. The rise in cardiac electronic device implantations in recent years underscores the significance of evaluating the quality of life among such patients.

Materials and methods: We conducted a study focusing on the quality of life of 438 patients with cardiac implantable electronic devices (cardiac pacemakers, cardioverter-defibrillators, cardiac resynchronization therapy devices). These patients were diagnosed with sick sinus syndrome, high-degree atrioventricular (AV) block, or severe heart failure (New York Heart Association (NYHA) classes III- IV (NYHA III-IV)), with left ventricular ejection fraction (LVEF) ≤ 35%, with/without complete left bundle branch block (QRS ≥ 130 μs), or with a history of ventricular tachycardia/ventricular fibrillation. The study utilized the EuroQol 5-Dimension 5-level (EQ-5D-5L) questionnaire and the EQ visual analog scale, which patients completed both prior to cardiac device implantation and during six post-implantation follow-up visits. The analysis of the research findings was conducted using the IBM SPSS Statistics software program (Armonk, NY).

Results: Cardiac pacemaker implantation in patients with sick sinus syndrome and high-grade AV block demonstrated significant and highly reliable positive effects on quality of life concerning mobility, self-care, and usual activity. Similarly, cardiac resynchronization device implantation in individuals with severe heart failure with reduced LVEF and wide QRS showed significant positive effects in these areas. However, cardioverter-defibrillator implantation did not yield positive effects on these modules. Regarding pain/discomfort, neither pacemaker nor cardiac resynchronization device implantation resulted in improved quality of life, while there was a somewhat positive effect observed in the cardioverter-defibrillator group. In terms of anxiety/depression, pacemaker implantation in patients with sick sinus syndrome and high-degree AV block had a significant and highly reliable positive impact on quality of life. Additionally, relatively positive impacts were noted at various periods following cardioverter-defibrillator and cardiac resynchronization device implantations.

Conclusions: Cardiac implantable electronic devices play a crucial role not only in saving lives but also in positively impacting the quality of life of patients when appropriately selected.

## Introduction

Over recent decades, advancements in medical technology have not only saved lives but also enhanced overall health and quality of life [1]. Within modern medicine, there has been a growing utilization of various types of cardiac implantable electronic devices (CIED), with their applications expanding continuously [2]. These cardiac devices play a crucial role in preventing sudden cardiac death due to rhythm disturbances and/or improving heart systolic function, thus impacting patients' quality of life to a certain extent [3]. Examining the quality of life across different stages of post-implantation remains a topical focus within the healthcare system [4].

Quality of life stands as a paramount measure to assess a patient's clinical status, treatment outcomes, and treatment effectiveness.

## Materials and methods

The research protocol underwent review and approval by the Medical Ethics Commission of the L. Sakvarelidze National Center for Disease Control and Public Health. The study was conducted at the G. Chapidze Emergency Cardiology Center, in Tbilisi, Georgia. We assessed the quality of life of male and female patients aged ≥ 18 who were either scheduled to undergo or had previously undergone implantation of cardiac electronic devices (cardiac pacemakers (PM), cardioverter-defibrillators (ICD), or cardiac resynchronization therapy devices (CRT-P/D)). Inclusion criteria for the study and data collection were implemented from December 12, 2018, to November 30, 2023.

Indications for pacemaker implantation included sick sinus syndrome or high-grade AV block. For cardioverter-defibrillators, indications were severe heart failure (NYHA III-IV) with significantly reduced left ventricular ejection fraction (LVEF ≤ 35%) or a history of ventricular tachycardia and/or ventricular fibrillation. Regarding cardiac resynchronization device implantation, this cardiac device was implanted in individuals with severe heart failure (NYHA III-IV) and significantly reduced left ventricular ejection fraction (LVEF ≤ 35%), accompanied by complete left bundle branch block (QRS ≥ 130 μs). It is noteworthy that, prior to the implantation of ICD and CRT-P/D for primary prevention, patients had been receiving optimal medical treatment recommended for chronic heart failure for more than three months.

A prerequisite for inclusion in the study was obtaining written informed consent from the individuals being researched. Exclusion criteria comprised individuals under 18 years of age and those who were not proficient in the Georgian language. Patients scheduled for cardiac device implantation completed the EQ-5D-5L questionnaire and the EQ visual analog scale before and after the procedure [5]. We obtained permission to use the EQ-5D-5L questionnaire and the EQ visual analog scale from the original publishers. The EQ-5D-5L questionnaire collected data on mobility, self-care, usual activity, pain/discomfort, and anxiety/depression. Subjects selected the appropriate response level for each item, selecting from options including "no problem," "slight problem," "moderate problem," "severe problem," or "extreme problem.” On the EQ visual analog scale, patients indicated a score determining their overall health status from 0 to 100, where 0 denoted the worst condition and 100 denoted the best. These questionnaires were utilized to gather data for the research across six visits. The first visit occurred prior to cardiac device implantation. The second visit took place 9-14 days post-CIED implantation. The third visit occurred 30-45 days post-implantation, the fourth visit at six months post-implantation, the fifth visit at one year post-implantation, and the sixth visit at three years post-implantation. For patients who already had a cardiac device implanted at the time of study inclusion, they completed the aforementioned questionnaires and provided information corresponding to the relevant timeframes. In addition to the aforementioned data, patient demographic information such as age and gender, as well as details on side effects and complications of cardiac device implantation, were collected as part of the study. The examination of the study's outcomes utilized the Statistical Product and Service Solutions (SPSS; IBM SPSS Statistics for Windows, Armonk, NY) software, applying both the chi-square test and the paired samples T-test for analysis.

## Results

A total of 438 patients were enrolled in the study. Among these patients, 110 were with sick sinus syndrome and PM implantation (males: n=66 (60%), females: n=44 (40%), age <65 y.o.: n=53 (48.2%), age ≥65 y.o.: n=57 (51.8%), 110 with high-grade AV block and PM implantation (males: n=48 (43.6%), females: n=62 (56.4%), age <65 y.o.: n=56 (50.9%), age ≥65 y.o.: n=54 (49.1%), 116 with ICD implantation (males: n=83 (71.6%), females: n=33 (28.4%), age <65 y.o.: n=78 (67.2%), age ≥65 y.o.: n=38 (32.8%) and 102 with CRT-P/D implantation (males: n=69 (67.6%), females: n=33 (32.4%), age <65 y.o.: n=56 (54.9%), age ≥ 65 y.o.: n=46 (45.1%).

Results of the study in patients with sick sinus syndrome and pacemaker implantation

Table 1 presents the results of the mobility module for patients with sick sinus syndrome and PM implantation across different visits.

**Table 1:** Results of the mobility module for patients with sick sinus syndrome and PM implantation Visit I - visit before the PM implantation, visit II - visit 9-14 days after the PM implantation, visit III - visit 30-45 days after the PM implantation, visit IV - visit six months after the PM implantation, visit V - visit one year after the PM implantation, visit VI - visit three years after the PM implantation

			Visit I	Visit II	Visit III	Visit IV	Visit V	Visit VI
MOBILITY	I have no problems in walking about	Count	15	17	29	38	50	56
		% within visit	13.6%	15.5%	26.4%	34.5%	45.5%	50.9%
	I have slight problems in walking about	Count	33	45	51	48	52	47
		% within visit	30.0%	40.9%	46.4%	43.6%	47.3%	42.7%
	I have moderate problems in walking about	Count	36	25	13	16	4	4
		% within visit	32.7%	22.7%	11.8%	14.5%	3.6%	3.6%
	I have severe problems in walking about	Count	24	22	16	8	4	3
		% within visit	21.8%	20.0%	14.5%	7.3%	3.6%	2.7%
	I am unable to walk about	Count	2	1	1	0	0	0
		% within visit	1.8%	0.9%	0.9%	0.0%	0.0%	0.0%
Total		Count	110	110	110	110	110	110
		% within visit	100.0%	100.0%	100.0%	100.0%	100.0%	100.0%

According to the chi-square test, there is a statistically significant association between mobility and the number of visits X2 (20, N=110) = 132.081 (P=0.000).

Table 2 presents the results of the self-care module across different visits for patients with sick sinus syndrome and PM implantation.

**Table 2:** Results of the self-care module for patients with sick sinus syndrome and PM implantation Visit I - visit before the PM implantation, visit II - visit 9-14 days after the PM implantation, visit III - visit 30-45 days after the PM implantation, visit IV - visit six months after the PM implantation, visit V - visit one year after the PM implantation, visit VI - visit three years after the PM implantation

			Visit I	Visit II	Visit III	Visit IV	Visit V	Visit VI
SELF-CARE	I have no problems washing or dressing myself	Count	13	15	30	35	46	49
		% within visit	11.8%	13.6%	27.3%	31.8%	41.8%	44.5%
	I have slight problems washing or dressing myself	Count	37	40	50	42	46	49
		% within visit	33.6%	36.4%	45.5%	38.2%	41.8%	44.5%
	I have moderate problems washing or dressing myself	Count	37	25	14	17	5	4
		% within visit	33.6%	22.7%	12.7%	15.5%	4.5%	3.6%
	I have severe problems washing or dressing myself	Count	21	29	15	16	13	8
		% within visit	19.1%	26.4%	13.6%	14.5%	11.8%	7.3%
	I am unable to wash or dress myself	Count	2	1	1	0	0	0
		% within visit	1.8%	0.9%	0.9%	0.0%	0.0%	0.0%
Total		Count	110	110	110	110	110	110
		% within visit	100.0%	100.0%	100.0%	100.0%	100.0%	100.0%

According to the chi-square test, there is a statistically significant association between self-care and the number of visits X2 (20, N=110) = 106.246 (P=0.000).

Table 3 presents the results of the usual activities module across different visits for patients with sick sinus syndrome and PM implantation.

**Table 3:** Results of the usual activities module for patients with sick sinus syndrome and PM implantation Visit I - visit before the PM implantation, visit II - visit 9-14 days after the PM implantation, visit III - visit 30-45 days after the PM implantation, visit IV - visit three months after the PM implantation, visit V - visit one year after the PM implantation, visit VI - visit three years after the PM implantation

			Visit I	Visit II	Visit III	Visit IV	Visit V	Visit VI
USUAL ACTIVITIES	I have no problems doing my usual activities	Count	15	17	28	36	50	55
		% within visit	13.6%	15.5%	25.5%	32.7%	45.5%	50.0%
	I have slight problems doing my usual activities	Count	34	44	50	49	50	47
		% within visit	30.9%	40.0%	45.5%	44.5%	45.5%	42.7%
	I have moderate problems doing my usual activities	Count	37	21	14	8	4	3
		% within visit	33.6%	19.1%	12.7%	7.3%	3.6%	2.7%
	I have severe problems doing my usual activities	Count	22	27	17	17	6	5
		% within visit	20.0%	24.5%	15.5%	15.5%	5.5%	4.5%
	I am unable to do my usual activities	Count	2	1	1	0	0	0
		% within visit	1.8%	0.9%	0.9%	0.0%	0.0%	0.0%
Total		Count	110	110	110	110	110	110
		% within visit	100.0%	100.0%	100.0%	100.0%	100.0%	100.0%

According to the chi-square test, there is a statistically significant association between activity and the number of visits X2 (20, N=110) = 132.200 (P=0.000).

Table 4 presents the results of the pain/discomfort module across different visits for patients with sick sinus syndrome and PM implantation.

**Table 4:** Results of the pain/discomfort module for patients with sick sinus syndrome and PM implantation Visit I - visit before the PM implantation, visit II - visit 9-14 days after the PM implantation, visit III - visit 30-45 days after the PM implantation, visit IV - visit six months after the PM implantation, visit V - visit one year after the PM implantation, visit VI - visit three years after the PM implantation

			Visit I	Visit II	Visit III	Visit IV	Visit V	Visit VI
PAIN/DISCOMFORT	I have no pain or discomfort I have extreme pain or discomfort	Count	60	53	54	61	56	56
		% within visit	54.5%	48.2%	49.1%	55.5%	50.9%	50.9%
	I have slight pain or discomfort	Count	33	45	47	44	50	50
		% within visit	30.0%	40.9%	42.7%	40.0%	45.5%	45.5%
	I have moderate pain or discomfort	Count	12	9	8	5	4	4
		% within visit	10.9%	8.2%	7.3%	4.5%	3.6%	3.6%
	I have severe pain or discomfort	Count	3	1	0	0	0	0
		% within visit	2.7%	0.9%	0.0%	0.0%	0.0%	0.0%
	I have extreme pain or discomfort	Count	2	2	1	0	0	0
		% within visit	1.8%	1.8%	0.9%	0.0%	0.0%	0.0%
Total		Count	110	110	110	110	110	110
		% within visit	100.0%	100.0%	100.0%	100.0%	100.0%	100.0%

The result of the chi-square test indicates that there is no significant association between the pain variable and the number of visits X2 (20, N=110) = 29.569 (P>0.05).

Table 5 presents the results of the anxiety/depression module across different visits for patients with sick sinus syndrome and PM implantation.

**Table 5:** Results of the anxiety/depression module for patients with sick sinus syndrome and PM implantation Visit I - visit before the PM implantation, visit II - visit 9-14 days after the PM implantation, visit III - visit 30-45 days after the PM implantation, visit IV - visit six months after the PM implantation, visit V - visit one year after the PM implantation, visit VI - visit three years after the PM implantation

			Visit I	Visit II	Visit III	Visit IV	Visit V	Visit VI
ANXIETY/DEPRESSION	I am not anxious or depressed	Count	2	23	60	70	76	82
		% within visit	1.8%	20.9%	54.5%	63.6%	69.1%	74.5%
	I am slightly anxious or depressed	Count	25	24	10	15	17	13
		% within visit	22.7%	21.8%	9.1%	13.6%	15.5%	11.8%
	I am moderately anxious or depressed	Count	32	24	23	12	10	8
		% within visit	29.1%	21.8%	20.9%	10.9%	9.1%	7.3%
	I am severely anxious or depressed	Count	39	30	12	8	3	3
		% within visit	35.5%	27.3%	10.9%	7.3%	2.7%	2.7%
	I am extremely anxious or depressed	Count	12	9	5	5	4	4
		% within visit	10.9%	8.2%	4.5%	4.5%	3.6%	3.6%
Total		Count	110	110	110	110	110	110
		% within visit	100.0%	100.0%	100.0%	100.0%	100.0%	100.0%

According to the chi-square test, there is a statistically significant association between the anxiety and depression variable and the number of visits X2 (20, N=110) = 215.791 (P=0.000).

As a result of paired samples T-test of EQ visual analog scale data, it is visible that visit I is significantly different from all the other visits, and the biggest difference between visits is given on the first and last visits, where participant’s mean EQ scores (M=74.48, SD=14.411) on the sixth visit turned out to be 20,57 points higher than the mean scores on their first visit (M=(53.91, SD=(10.744)). t((109)) = (-17.244) (P=< 0.001; Table 6).

**Table 6:** Result of paired sample T-test of EQ visual analog scale data for patients with sick sinus syndrome and PM implantation (i) EQ1 - EQ visual analog scale data at visit I, before the PM implantation, EQ2 - EQ visual analog scale data at visit II, 9-14 days after the PM implantation, EQ3 - EQ visual analog scale data at visit III, 30-45 days after the PM implantation, EQ4 - EQ visual analog scale data at visit IV, six months after the PM implantation, EQ5 - EQ visual analog scale data at visit V, one year after the PM implantation, EQ6 - EQ visual analog scale data at visit VI, three years after the PM implantation (ii) Pair 1 - a comparison of visits I and II, pair 2 - a comparison of visits I and III, pair 3 - a comparison of visits I and IV, pair 4 - a comparison of visits I and V, pair 5 - a comparison of visits I and VI (iii) The total range of the EQ visual analog scale is between 0 and 100.

		Mean	N	Std. Deviation	Std. Error Mean
Pair 1	EQ1	53.9091	110	10.74350	1.02435
	EQ2	61.2091	110	13.46970	1.28429
Pair 2	EQ1	53.9091	110	10.74350	1.02435
	EQ3	66.5727	110	14.40468	1.37343
Pair 3	EQ1	53.9091	110	10.74350	1.02435
	EQ4	71.1091	110	13.38584	1.27629
Pair 4	EQ1	53.9091	110	10.74350	1.02435
	EQ5	73.6818	110	13.97175	1.33215
Pair 5	EQ1	53.9091	110	10.74350	1.02435
	EQ6	74.4818	110	14.41058	1.37399

Results of the study in patients with high-grade AV block and pacemaker implantation

Table 7 presents the results of the mobility module across different visits for patients with high-grade AV block and PM implantation.

**Table 7:** Results of the mobility module for patients with high-grade AV block and PM implantation Visit I - visit before the PM implantation, visit II - visit 9-14 days after the PM implantation, visit III - visit 30-45 days after the PM implantation, visit IV - visit six months after the PM implantation, visit V - visit one year after the PM implantation, visit VI - visit three years after the PM implantation

			Visit I	Visit II	Visit III	Visit IV	Visit V	Visit VI
MOBILITY	I have no problems in walking about	Count	1	31	45	55	57	56
		% within visit	0.9%	28.2%	40.9%	50.0%	51.8%	50.9%
	I have slight problems in walking about	Count	5	48	48	45	44	47
		% within visit	4.5%	43.6%	43.6%	40.9%	40.0%	42.7%
	I have moderate problems in walking about	Count	44	21	10	5	6	4
		% within visit	40.0%	19.1%	9.1%	4.5%	5.5%	3.6%
	I have severe problems in walking about	Count	50	8	6	5	3	3
		% within visit	45.5%	7.3%	5.5%	4.5%	2.7%	2.7%
	I am unable to walk about	Count	10	2	1	0	0	0
		% within visit	9.1%	1.8%	0.9%	0.0%	0.0%	0.0%
Total		Count	110	110	110	110	110	110
		% within visit	100.0%	100.0%	100.0%	100.0%	100.0%	100.0%

According to the chi-square test, there is a statistically significant association between mobility and the number of visits X2 (20, N=110) = 347.262 (P=0.000).

Table 8 presents the results of the self-care module across different visits for patients with high-grade AV block and PM implantation.

**Table 8:** Results of the self-care module for patients with high-grade AV block and PM implantation Visit I - visit before the PM implantation, visit II - visit 9-14 days after the PM implantation, visit III - visit 30-45 days after the PM implantation, visit IV - visit six months after the PM implantation, visit V - visit one year after the PM implantation, visit VI - visit three years after the PM implantation

			Visit I	Visit II	Visit III	Visit IV	Visit V	Visit VI
SELF-CARE	I have no problems washing or dressing myself	Count	2	30	47	52	60	62
		% within visit	1.8%	27.3%	42.7%	47.3%	54.5%	56.4%
	I have slight problems washing or dressing myself	Count	4	47	49	40	41	43
		% within visit	3.6%	42.7%	44.5%	36.4%	37.3%	39.1%
	I have moderate problems washing or dressing myself	Count	39	21	6	12	5	2
		% within visit	35.5%	19.1%	5.5%	10.9%	4.5%	1.8%
	I have severe problems washing or dressing myself	Count	52	9	6	6	4	3
		% within visit	47.3%	8.2%	5.5%	5.5%	3.6%	2.7%
	I am unable to wash or dress myself	Count	13	3	2	0	0	0
		% within visit	11.8%	2.7%	1.8%	0.0%	0.0%	0.0%
Total		Count	110	110	110	110	110	110
		% within visit	100.0%	100.0%	100.0%	100.0%	100.0%	100.0%

According to the chi-square test, there is a statistically significant association between self-care and the number of visits X2 (20, N=110) = 345.875 (P=0.000).

Table 9 presents the results of the usual activities module across different visits for patients with high-grade AV block and PM implantation.

**Table 9:** Results of the usual activities module for patients with high-grade AV block and PM implantation Visit I - visit before the PM implantation, visit II - visit 9-14 days after the PM implantation, visit III - visit 30-45 days after the PM implantation, visit IV - visit six months after the PM implantation, visit V - visit one year after the PM implantation, visit VI - visit three years after the PM implantation

			Visit I	Visit II	Visit III	Visit IV	Visit V	Visit VI
USUAL ACTIVITIES	I have no problems doing my usual activities	Count	1	29	47	50	62	62
		% within visit	0.9%	26.4%	42.7%	45.5%	56.4%	56.4%
	I have slight problems doing my usual activities	Count	3	46	48	41	40	40
		% within visit	2.7%	41.8%	43.6%	37.3%	36.4%	36.4%
	I have moderate problems doing my usual activities	Count	40	20	6	12	3	4
		% within visit	36.4%	18.2%	5.5%	10.9%	2.7%	3.6%
	I have severe problems doing my usual activities	Count	55	12	6	6	4	4
		% within visit	50.0%	10.9%	5.5%	5.5%	3.6%	3.6%
	I am unable to do my usual activities	Count	11	3	3	1	1	0
		% within visit	10.0%	2.7%	2.7%	0.9%	0.9%	0.0%
Total		Count	110	110	110	110	110	110
		% within visit	100.0%	100.0%	100.0%	100.0%	100.0%	100.0%

According to the chi-square test, there is a statistically significant association between activity and the number of visits X2 (20, N=110) = 338.606 (P=0.000).

Table 10 presents the results of the pain/discomfort module across different visits for patients with high-grade AV block and PM implantation.

**Table 10:** Results of the pain/discomfort module for patients with high-grade AV block and PM implantation Visit I - visit before the PM implantation, visit II - visit 9-14 days after the PM implantation, visit III - visit 30-45 days after the PM implantation, visit IV - visit six months after the PM implantation, visit V - visit one year after the PM implantation, visit VI - visit three years after the PM implantation

			Visit I	Visit II	Visit III	Visit IV	Visit V	Visit VI
PAIN/DISCOMFORT	I have no pain or discomfort I have extreme pain or discomfort	Count	55	59	54	61	71	72
		% within visit	50.0%	53.6%	49.1%	55.5%	64.5%	65.5%
	I have slight pain or discomfort	Count	37	39	48	42	29	28
		% within visit	33.6%	35.5%	43.6%	38.2%	26.4%	25.5%
	I have moderate pain or discomfort	Count	10	9	6	4	7	8
		% within visit	9.1%	8.2%	5.5%	3.6%	6.4%	7.3%
	I have severe pain or discomfort	Count	5	1	1	2	1	1
		% within visit	4.5%	0.9%	0.9%	1.8%	0.9%	0.9%
	I have extreme pain or discomfort	Count	3	2	1	1	2	1
		% within visit	2.7%	1.8%	0.9%	0.9%	1.8%	0.9%
Total		Count	110	110	110	110	110	110
		% within visit	100.0%	100.0%	100.0%	100.0%	100.0%	100.0%

The result of the chi-square test indicates that there is no significant association between the pain variable and the number of visits X2 (20, N=110) = 25.018 (P>0.05).

Table 11 presents the results of the anxiety/depression module across different visits for patients with high-grade AV block and PM implantation.

**Table 11:** Results of the anxiety/depression module for patients with high-grade AV block and PM implantation Visit I - visit before the PM implantation, visit II - visit 9-14 days after the PM implantation, visit III - visit 30-45 days after the PM implantation, visit IV - visit six months after the PM implantation, visit V - visit one year after the PM implantation, visit VI - visit three years after the PM implantation

			Visit I	Visit II	Visit III	Visit IV	Visit V	Visit VI
ANXIETY/DEPRESSION	I am not anxious or depressed	Count	5	26	72	71	76	84
		% within visit	4.5%	23.6%	65.5%	64.5%	69.1%	76.4%
	I am slightly anxious or depressed	Count	11	32	21	27	24	17
		% within visit	10.0%	29.1%	19.1%	24.5%	21.8%	15.5%
	I am moderately anxious or depressed	Count	43	37	3	5	7	6
		% within visit	39.1%	33.6%	2.7%	4.5%	6.4%	5.5%
	I am severely anxious or depressed	Count	35	12	11	1	1	1
		% within visit	31.8%	10.9%	10.0%	0.9%	0.9%	0.9%
	I am extremely anxious or depressed	Count	16	3	3	6	2	2
		% within visit	14.5%	2.7%	2.7%	5.5%	1.8%	1.8%
Total		Count	110	110	110	110	110	110
		% within visit	100.0%	100.0%	100.0%	100.0%	100.0%	100.0%

According to the chi-square test, there is a statistically significant association between the anxiety and depression variable and the number of visits X2 (20, N=110) = 316.051 (P=0.000).

As a result of paired sample T-test of EQ visual analog scale data, it is visible that visit N1 is significantly different from all the other visits, and the biggest difference between visits is given on the first and last visits, where participant’s mean EQ scores (M=75.45, SD=13.000) on the sixth visit turned out to be 24,73 points higher than the mean scores on their first visit (M=50.71, SD=(14.411). t((109)) = (-18.904) (P=< 0.001; Table 12).

**Table 12:** Result of paired samples T-test of EQ visual analog scale data for patients with high-grade AV block and PM implantation (i) EQ1 - EQ visual analog scale data at visit I, before the PM implantation, EQ2 - EQ visual analog scale data at visit II, 9-14 days after the PM implantation, EQ3 - EQ visual analog scale data at visit III, 30-45 days after the PM implantation, EQ4 - EQ visual analog scale data at visit IV, six months after the PM implantation, EQ5 - EQ visual analog scale data at visit V, one year after the PM implantation, EQ6 - EQ visual analog scale data at visit VI, three years after the PM implantation (ii) Pair 1 - a comparison of visits I and II, pair 2 - a comparison of visits I and III, pair 3 - a comparison of visits I and IV, pair 4 - a comparison of visits I and V, pair 5 - a comparison of visits I and VI (iii) The total range of the EQ visual analog scale is between 0 and 100.

		Mean	N	Std. Deviation	Std. Error Mean
Pair 1	EQ1	50.7182	110	10.19366	0.97193
	EQ2	65.6909	110	14.86190	1.41703
Pair 2	EQ1	50.7182	110	10.19366	0.97193
	EQ3	70.3545	110	13.41681	1.27924
Pair 3	EQ1	50.7182	110	10.19366	0.97193
	EQ4	74.4727	110	12.24348	1.16737
Pair 4	EQ1	50.7182	110	10.19366	0.97193
	EQ5	74.9636	110	12.48260	1.19017
Pair 5	EQ1	50.7182	110	10.19366	0.97193
	EQ6	75.4545	110	13.00010	1.23951

Results of the study in patients with ICD implantation

Table 13 presents the results of the mobility module across different visits for patients who underwent ICD implantation.

**Table 13:** Results of the mobility module for patients with ICD implantation Visit I - visit before the ICD implantation, visit II - visit 9-14 days after the ICD implantation, visit III - visit 30-45 days after the ICD implantation, visit IV - visit six months after the ICD implantation, visit V - visit one year after the ICD implantation, visit VI - visit three years after the ICD implantation

			Visit I	Visit II	Visit III	Visit IV	Visit V	Visit VI
MOBILITY	I have no problems in walking about	Count	9	10	9	10	10	10
		% within visit	7.8%	8.6%	7.8%	8.6%	8.6%	8.6%
	I have slight problems in walking about	Count	12	14	15	14	12	12
		% within visit	10.3%	12.1%	12.9%	12.1%	10.3%	10.3%
	I have moderate problems in walking about	Count	35	37	32	28	25	25
		% within visit	30.2%	31.9%	27.6%	24.1%	21.6%	21.6%
	I have severe problems in walking about	Count	60	55	60	64	66	65
		% within visit	51.7%	47.4%	51.7%	55.2%	56.9%	56.0%
	I am unable to walk about	Count	0	0	0	0	3	4
		% within visit	0.0%	0.0%	0.0%	0.0%	2.6%	3.4%
Total		Count	116	116	116	116	116	116
		% within visit	100.0%	100.0%	100.0%	100.0%	100.0%	100.0%

According to the chi-square test, there is a statistically significant association between mobility and the number of visits X2 (20, N=110) = 347.262 (P=0.000).

Table 14 presents the results of the self-care module across different visits for patients who underwent ICD implantation.

**Table 14:** Results of the self-care module for patients with ICD implantation Visit I - visit before the ICD implantation, visit II - visit 9-14 days after the ICD implantation, visit III - visit 30-45 days after the ICD implantation, visit IV - visit 6 months after the ICD implantation, visit V - visit 1 year after the ICD implantation, visit VI - visit 3 years after the ICD implantation.

			Visit I	Visit II	Visit III	Visit IV	Visit V	Visit VI
SELF-CARE	I have no problems washing or dressing myself	Count	12	13	13	15	15	13
		% within visit	10.3%	11.2%	11.2%	12.9%	12.9%	11.2%
	I have slight problems washing or dressing myself	Count	12	14	15	15	12	12
		% within visit	10.3%	12.1%	12.9%	12.9%	10.3%	10.3%
	I have moderate problems washing or dressing myself	Count	36	39	38	33	34	32
		% within visit	31.0%	33.6%	32.8%	28.4%	29.3%	27.6%
	I have severe problems washing or dressing myself	Count	56	50	50	53	51	55
		% within visit	48.3%	43.1%	43.1%	45.7%	44.0%	47.4%
	I am unable to wash or dress myself	Count	0	0	0	0	4	4
		% within visit	0.0%	0.0%	0.0%	0.0%	3.4%	3.4%
Total		Count	116	116	116	116	116	116
		% within visit	100.0%	100.0%	100.0%	100.0%	100.0%	100.0%

According to the chi-square test, there is no statistically significant association between self-care and the number of visits X2 (20, N=116) = 19.157 (P=0.05).

Table 15 presents the results of the usual activities module across different visits for patients who underwent ICD implantation.

**Table 15:** Results of the usual activities module for patients with ICD implantation Visit I - visit before the ICD implantation, visit II - visit 9-14 days after the ICD implantation, visit III - visit 30-45 days after the ICD implantation, visit IV - visit six months after the ICD implantation, visit V - visit one year after the ICD implantation, visit VI - visit three years after the ICD implantation

			Visit I	Visit II	Visit III	Visit IV	Visit V	Visit VI
USUAL ACTIVITIES	I have no problems doing my usual activities	Count	8	9	9	10	10	10
		% within visit	6.9%	7.8%	7.8%	8.6%	8.6%	8.6%
	I have slight problems doing my usual activities	Count	14	15	15	17	15	14
		% within visit	12.1%	12.9%	12.9%	14.7%	12.9%	12.1%
	I have moderate problems doing my usual activities	Count	36	38	40	41	39	40
		% within visit	31.0%	32.8%	34.5%	35.3%	33.6%	34.5%
	I have severe problems doing my usual activities	Count	58	54	51	46	47	46
		% within visit	50.0%	46.6%	44.0%	39.7%	40.5%	39.7%
	I am unable to do my usual activities	Count	0	0	1	2	5	6
		% within visit	0.0%	0.0%	0.9%	1.7%	4.3%	5.2%
Total		Count	116	116	116	116	116	116
		% within visit	100.0%	100.0%	100.0%	100.0%	100.0%	100.0%

According to the chi-square test, there is no statistically significant association between activity and number of visits X2 (20, N=116) = 17.864 (P>0.05).

Table 16 presents the results of the pain/discomfort module across different visits for patients who underwent ICD implantation.

**Table 16:** Results of the pain/discomfort module for patients with ICD implantation Visit I - visit before the ICD implantation, visit II - visit 9-14 days after the ICD implantation, visit III - visit 30-45 days after the ICD implantation, visit IV - visit six months after the ICD implantation, visit V - visit one year after the ICD implantation, visit VI - visit three years after the ICD implantation

			Visit I	Visit II	Visit III	Visit IV	Visit V	Visit VI
PAIN/DISCOMFORT	I have no pain or discomfort I have extreme pain or discomfort	Count	65	88	95	99	99	97
		% within visit	56.0%	75.9%	81.9%	85.3%	85.3%	83.6%
	I have slight pain or discomfort	Count	44	23	19	17	17	17
		% within visit	37.9%	19.8%	16.4%	14.7%	14.7%	14.7%
	I have moderate pain or discomfort	Count	7	5	2	0	0	0
		% within visit	6.0%	4.3%	1.7%	0.0%	0.0%	0.0%
	I have severe pain or discomfort	Count	0	0	0	0	0	2
		% within visit	0.0%	0.0%	0.0%	0.0%	0.0%	1.7%
	I have extreme pain or discomfort	Count	0	0	0	0	0	0
		% within visit	0.0%	0.0%	0.0%	0.0%	0.0%	0.0%
Total		Count	116	116	116	116	116	116
		% within visit	100.0%	100.0%	100.0%	100.0%	100.0%	100.0%

The result of the chi-square test indicates that there is a significant association between the pain variable and the number of visits X2 (20, N=116) = 63.707 (P=0.000).

Table 17 presents the results of the anxiety/depression module across different visits for patients who underwent ICD implantation.

**Table 17:** Results of the anxiety/depression module for patients with ICD implantation Visit I - visit before the ICD implantation, visit II - visit 9-14 days after the ICD implantation, visit III - visit 30-45 days after the ICD implantation, visit IV - visit six months after the ICD implantation, visit V - visit one year after the ICD implantation, visit VI - visit three years after the ICD implantation

			Visit I	Visit II	Visit III	Visit IV	Visit V	Visit VI
ANXIETY/DEPRESSION	I am not anxious or depressed	Count	7	4	0	0	0	0
		% within visit	6.0%	3.4%	0.0%	0.0%	0.0%	0.0%
	I am slightly anxious or depressed	Count	12	20	68	71	73	70
		% within visit	10.3%	17.2%	58.6%	61.2%	62.9%	60.3%
	I am moderately anxious or depressed	Count	84	78	32	28	27	28
		% within visit	72.4%	67.2%	27.6%	24.1%	23.3%	24.1%
	I am severely anxious or depressed	Count	10	12	14	15	14	15
		% within visit	8.6%	10.3%	12.1%	12.9%	12.1%	12.9%
	I am extremely anxious or depressed	Count	3	2	2	2	2	3
		% within visit	2.6%	1.7%	1.7%	1.7%	1.7%	2.6%
Total		Count	116	116	116	116	116	116
		% within visit	100.0%	100.0%	100.0%	100.0%	100.0%	100.0%

According to the chi-square test, there is a statistically significant association between the anxiety and depression variable and the number of visits X2 (20, N=116) = 182.567 (P=0.000).

As a result of the paired samples T-test of EQ visual analog scale data, it is visible that visit N1 is significantly different from visits N2, N3, and N6. The biggest difference between visits is given on the first and last visits, where the participant’s mean EQ scores (M=38.56, SD=11.800) on the sixth visit turned out to be 1.974 points lower than the mean scores on their first visit (M=(40.53), SD=(12.322)). t((109)) = (3.010) (P=<0.05; Table 18).

**Table 18:** Result of the paired sample T-test of EQ visual analog scale data for patients with ICD implantation (i) EQ1 - EQ visual analog scale data at visit I, before the ICD implantation, EQ2 - EQ visual analog scale data at visit II, 9-14 days after the ICD implantation, EQ3 - EQ visual analog scale data at visit III, 30-45 days after the ICD implantation, EQ4 - EQ visual analog scale data at visit IV, six months after the ICD implantation, EQ5 - EQ visual analog scale data at visit V, one year after the ICD implantation, EQ6 - EQ visual analog scale data at visit VI, three years after the ICD implantation (ii) Pair 1 - a comparison of visits I and II, pair 2 - a comparison of visits I and III, pair 3 - a comparison of visits I and IV, pair 4 - a comparison of visits I and V, pair 5 - a comparison of visits I and VI (iii) The total range of the EQ visual analog scale is between 0 and 100.

		Mean	N	Std. Deviation	Std. Error Mean
Pair 1	EQ1	40.5345	116	12.32490	1.14434
	EQ2	41.5690	116	12.54642	1.16491
Pair 2	EQ1	40.5345	116	12.32490	1.14434
	EQ3	42.0259	116	12.73712	1.18261
Pair 3	EQ1	40.5345	116	12.32490	1.14434
	EQ4	40.5603	116	12.63693	1.17331
Pair 4	EQ1	40.5345	116	12.32490	1.14434
	EQ5	39.8190	116	12.04961	1.11878
Pair 5	EQ1	40.5345	116	12.32490	1.14434
	EQ6	38.5603	116	11.80736	1.09629

Results of the study in patients with CRT-P/D implantation

Table 19 presents the results of the mobility module across different visits for patients who underwent CRT-P/D implantation.

**Table 19:** Results of the mobility module for patients with CRT-P/D implantation Visit I - visit before the CRT-P/D implantation, visit II - visit 9-14 days after the CRT-P/D implantation, visit III - visit 30-45 days after the CRT-P/D implantation, visit IV - visit six months after the CRT-P/D implantation, visit V - visit one year after the CRT-P/D implantation, visit VI - visit three years after the CRT-P/D implantation

			Visit I	Visit II	Visit III	Visit IV	Visit V	Visit VI
MOBILITY	I have no problems in walking about	Count	0	0	15	20	23	21
		% within visit	0.0%	0.0%	14.7%	19.6%	22.5%	20.6%
	I have slight problems in walking about	Count	0	0	24	38	35	36
		% within visit	0.0%	0.0%	23.5%	37.3%	34.3%	35.3%
	I have moderate problems in walking about	Count	5	13	36	17	18	20
		% within visit	4.9%	12.7%	35.3%	16.7%	17.6%	19.6%
	I have severe problems in walking about	Count	85	80	25	26	25	24
		% within visit	83.3%	78.4%	24.5%	25.5%	24.5%	23.5%
	I am unable to walk about	Count	12	9	2	1	1	1
		% within visit	11.8%	8.8%	2.0%	1.0%	1.0%	1.0%
Total		Count	102	102	102	102	102	102
		% within visit	100.0%	100.0%	100.0%	100.0%	100.0%	100.0%

According to the chi-square test, there is a statistically significant association between mobility and the number of visits X2 (20, N=102) = 270.454 (P=0.000).

Table 20 presents the results of the self-care module across different visits for patients who underwent CRT-P/D implantation.

**Table 20:** Results of the self-care module for patients with CRT-P/D implantation Visit I - visit before the CRT-P/D implantation, visit II - visit 9-14 days after the CRT-P/D implantation, visit III - visit 30-45 days after the CRT-P/D implantation, visit IV - visit six months after the CRT-P/D implantation, visit V - visit one year after the CRT-P/D implantation, visit VI - visit three years after the CRT-P/D implantation

			Visit I	Visit II	Visit III	Visit IV	Visit V	Visit VI
SELF-CARE	I have no problems washing or dressing myself	Count	0	0	13	20	22	20
		% within visit	0.0%	0.0%	12.7%	19.6%	21.6%	19.6%
	I have slight problems washing or dressing myself	Count	0	0	28	36	34	31
		% within visit	0.0%	0.0%	27.5%	35.3%	33.3%	30.4%
	I have moderate problems washing or dressing myself	Count	7	14	26	17	19	27
		% within visit	6.9%	13.7%	25.5%	16.7%	18.6%	26.5%
	I have severe problems washing or dressing myself	Count	83	78	25	27	26	23
		% within visit	81.4%	76.5%	24.5%	26.5%	25.5%	22.5%
	I am unable to wash or dress myself	Count	12	10	10	2	1	1
		% within visit	11.8%	9.8%	9.8%	2.0%	1.0%	1.0%
Total		Count	102	102	102	102	102	102
		% within visit	100.0%	100.0%	100.0%	100.0%	100.0%	100.0%

According to the chi-square test, there is a statistically significant association between self-care and the number of visits X2 (20, N=102) = 238.932 (P=0.000).

Table 21 presents the results of the usual activities module across different visits for patients who underwent CRT-P/D implantation.

**Table 21:** Results of the usual activities module for patients with CRT-P/D implantation Visit I - visit before the CRT-P/D implantation, visit II - visit 9-14 days after the CRT-P/D implantation, visit III - visit 30-45 days after the CRT-P/D implantation, visit IV - visit six months after the CRT-P/D implantation, visit V - visit one year after the CRT-P/D implantation, visit VI - visit three years after the CRT-P/D implantation

			Visit I	Visit II	Visit III	Visit IV	Visit V	Visit VI
USUAL ACTIVITIES	I have no problems doing my usual activities	Count	0	0	15	21	22	22
		% within visit	0.0%	0.0%	14.7%	20.6%	21.6%	21.6%
	I have slight problems doing my usual activities	Count	0	0	27	31	33	32
		% within visit	0.0%	0.0%	26.5%	30.4%	32.4%	31.4%
	I have moderate problems doing my usual activities	Count	7	15	23	19	20	23
		% within visit	6.9%	14.7%	22.5%	18.6%	19.6%	22.5%
	I have severe problems doing my usual activities	Count	83	77	28	28	26	24
		% within visit	81.4%	75.5%	27.5%	27.5%	25.5%	23.5%
	I am unable to do my usual activities	Count	12	10	9	3	1	1
		% within visit	11.8%	9.8%	8.8%	2.9%	1.0%	1.0%
Total		Count	102	102	102	102	102	102
		% within visit	100.0%	100.0%	100.0%	100.0%	100.0%	100.0%

According to the chi-square test, there is a statistically significant association between activity and number of visits X2 (20, N=102) = 222.164 (P=0.000).

Table 22 presents the results of the pain/discomfort module across different visits for patients who underwent CRT-P/D implantation.

**Table 22:** Results of the pain/discomfort module for patients with CRT-P/D implantation Visit I - visit before the CRT-P/D implantation, visit II - visit 9-14 days after the CRT-P/D implantation, visit III - visit 30-45 days after the CRT-P/D implantation, visit IV - visit six months after the CRT-P/D implantation, visit V - visit one year after the CRT-P/D implantation, visit VI - visit three years after the CRT-P/D implantation

			Visit I	Visit II	Visit III	Visit IV	Visit V	Visit VI
PAIN/DISCOMFORT	I have no pain or discomfort I have extreme pain or discomfort	Count	58	1	1	55	57	53
		% within visit	56.9%	1.0%	1.0%	53.9%	55.9%	52.0%
	I have slight pain or discomfort	Count	31	29	42	35	35	38
		% within visit	30.4%	28.4%	41.2%	34.3%	34.3%	37.3%
	I have moderate pain or discomfort	Count	6	62	52	7	4	6
		% within visit	5.9%	60.8%	51.0%	6.9%	3.9%	5.9%
	I have severe pain or discomfort	Count	5	7	5	4	4	4
		% within visit	4.9%	6.9%	4.9%	3.9%	3.9%	3.9%
	I have extreme pain or discomfort	Count	2	3	2	1	2	1
		% within visit	2.0%	2.9%	2.0%	1.0%	2.0%	1.0%
Total		Count	102	102	102	102	102	102
		% within visit	100.0%	100.0%	100.0%	100.0%	100.0%	100.0%

The result of the chi-square test indicates that there is a significant association between the pain variable and the number of visits X2 (20, N=102) = 268.849 (P=0.000).

Table 23 presents the results of the anxiety/depression module across different visits for patients who underwent CRT-P/D implantation.

**Table 23:** Results of the anxiety/depression module for patients with CRT-P/D implantation Visit I - visit before the CRT-P/D implantation, visit II - visit 9-14 days after the CRT-P/D implantation, visit III - visit 30-45 days after the CRT-P/D implantation, visit IV - visit six months after the CRT-P/D implantation, visit V - visit one year after the CRT-P/D implantation, visit VI - visit three years after the CRT-P/D implantation

			Visit I	Visit II	Visit III	Visit IV	Visit V	Visit VI
ANXIETY/DEPRESSION	I am not anxious or depressed	Count	2	0	0	5	3	3
		% within visit	2.0%	0.0%	0.0%	4.9%	2.9%	2.9%
	I am slightly anxious or depressed	Count	5	4	4	22	25	25
		% within visit	4.9%	3.9%	3.9%	21.6%	24.5%	24.5%
	I am moderately anxious or depressed	Count	64	49	38	54	53	53
		% within visit	62.7%	48.0%	37.3%	52.9%	52.0%	52.0%
	I am severely anxious or depressed	Count	18	32	30	10	11	11
		% within visit	17.6%	31.4%	29.4%	9.8%	10.8%	10.8%
	I am extremely anxious or depressed	Count	13	17	30	11	10	10
		% within visit	12.7%	16.7%	29.4%	10.8%	9.8%	9.8%
Total		Count	102	102	102	102	102	102
		% within visit	100.0%	100.0%	100.0%	100.0%	100.0%	100.0%

According to the chi-square test, there is a statistically significant association between the anxiety and depression variable and the number of visits X2 (20, N=102) = 103.415 (P=0.000).

As a result of paired sample T-test of EQ visual analog scale data, it is visible that Visit N1 is significantly different from all the other visits, and the biggest difference between visits is given on the first and last visits, where participant’s mean EQ scores (M=62.34, SD=18,588) on the sixth visit turned out to be 28.60 points higher than the mean scores on their first visit (M=(33.73), SD=(10,640)). t((109)) = (-17.356) (P=<0.001; Table 24).

**Table 24:** Result of paired sample T-test of EQ visual analog scale data for patients with CRT-P/D implantation (i) EQ1 - EQ visual analog scale data at visit I, before the CRT-P/D implantation, EQ2 - EQ visual analog scale data at visit II, 9-14 days after the CRT-P/D implantation, EQ3 - EQ visual analog scale data at visit III, 30-45 days after the CRT-P/D implantation, EQ4 - EQ visual analog scale data at visit IV, six months after the CRT-P/D implantation, EQ5 - EQ visual analog scale data at visit V, one year after the CRT-P/D implantation, EQ6 - EQ visual analog scale data at visit VI, three years after the CRT-P/D implantation (ii) Pair 1 - a comparison of visits I and II, pair 2 - a comparison of visits I and III, pair 3 - a comparison of visits I and IV, pair 4 - a comparison of visits I and V, pair 5 - a comparison of visits I and VI (iii) The total range of the EQ visual analog scale is between 0 and 100.

		Mean	N	Std. Deviation	Std. Error Mean
Pair 1	EQ1	33.7353	102	10.64357	1.05387
	EQ2	35.3235	102	11.42965	1.13170
Pair 2	EQ1	33.7353	102	10.64357	1.05387
	EQ3	63.5196	102	17.98720	1.78100
Pair 3	EQ1	33.7353	102	10.64357	1.05387
	EQ4	64.6961	102	18.32479	1.81442
Pair 4	EQ1	33.7353	102	10.64357	1.05387
	EQ5	64.5294	102	19.30101	1.91108
Pair 5	EQ1	33.7353	102	10.64357	1.05387
	EQ6	62.3431	102	18.58323	1.84001

Regarding age and gender, the study revealed that these variables do not exert a noteworthy influence on any of the modules utilized for evaluating the quality of life among patients undergoing cardiac device implantation. Concerning complications, the study identified the subsequent procedural complications: coronary sinus dissection in a single instance, pneumothorax in four instances, dislodgment of the implanted lead in three instances, and subcutaneous pocket infection in eight instances. The mortality during the procedure period was not observed.

## Discussion

Our study involving subjects with various diagnoses revealed fluctuations in the quality of life at different intervals following cardiac electronic device implantation. In the mobility module, patients with sick sinus syndrome who underwent pacemaker implantation experienced a reduction in walking difficulties starting from the second visit, showing continuous improvement throughout the study period. The data obtained are particularly noteworthy in the self-care module, where significant symptomatic improvement was observed among individuals struggling with washing or dressing. From the second visit onwards, there was a notable increase in the number of subjects reporting complete resolution of these complaints during self-care activities. This is likely attributed to the appropriate selection of patients for pacemaker implantation and the early positive impact of the cardiac device on normalizing heart rate. Furthermore, significant positive benefits were observed in the usual activity module, indicating enhanced physical activity and reduced associated problems in patients shortly after pacemaker implantation, a trend that persisted in the later stages of the study. However, our findings differ somewhat from a previous study, which reported a significant improvement in quality of life at four months post-implantation, followed by a reversal at six months after device implantation [6]. Regarding the pain/discomfort module, our study indicated that pacemaker implantation did not yield significant improvements in this aspect for individuals with sick sinus syndrome. In terms of the anxiety/depression module, subjective improvement was noted in patients at all visits following pacemaker implantation, likely attributed to the anxiety induced by bradycardia in sick sinus syndrome or, in some cases, depression [7]. The positive effects of cardiac electronic device implantation are likely due to the elimination of bradycardia and normalization of heart rate. However, previous studies have observed episodes of anxiety in this patient group, often associated with the size of the cardiac device and post-procedural wound pain [8].

Our study also sheds light on the impact of pacemaker implantation on the quality of life of subjects diagnosed with a high-grade AV block. In this cohort, pacemaker implantation exhibited a positive effect on quality of life. We studied both early and late post-implantation periods and observed a significant improvement in mobility, self-care, and usual activity among these individuals. However, regarding the pain/discomfort module, the implantation of a cardiac device did not yield a clinically significant impact on the subjects likely because pain/discomfort in this population is not primarily associated with AV conduction disorders. In contrast, our findings diverge in terms of anxiety/depression. It appears that anxiety/depression in individuals with high-grade AV block is largely attributable to AV conduction disorders. This assertion is supported by the observation of a positive impact on the mental health of subjects shortly after CIED implantation, a trend that persisted throughout the study period, including the final stage.

Our study yielded intriguing insights into the quality of life of subjects who underwent cardioverter-defibrillator implantation. Analysis of data from the mobility, self-care, and usual activity modules suggests that the implantation of a cardioverter-defibrillator does not notably impact aspects of quality of life requiring physical exertion. This observation can be attributed to the fact that these devices are typically implanted in individuals diagnosed with or at risk of life-threatening arrhythmias, such as ventricular tachycardia and/or ventricular fibrillation. The primary function of a cardioverter-defibrillator is to intervene actively in cases when ventricular tachycardia or ventricular fibrillation develops. Changes observed in mobility, self-care, and usual activity indicators across visits may be attributed to subjects' adherence to medical treatment and their underlying diagnosis, particularly heart failure. Symptoms were experienced as a result of possible decompensation of different degrees of this diagnosis during the visits. Our findings are consistent with those of the SCD-HeFT study, which reported no clinically or statistically significant differences in long-term quality of life improvement between the ICD group and the control group at 30 months post-implantation [9]. Similar results were also observed in the statistically highly reliable DEFINITE and MADIT II studies [10,11]. The data obtained in our study regarding the pain/discomfort module were particularly interesting. We observed a decrease in the rate of mild pain/discomfort starting from the second study visit, accompanied by an increase in the proportion of subjects reporting no pain/discomfort. It is important to note that the function of the cardioverter-defibrillator does not directly impact this parameter. The observed result is likely due to patients feeling safe post-ICD implantation, suggesting a psychological aspect contributing to the decrease in pain/discomfort rates. This interpretation is supported by data from the anxiety/depression module, which indicated a decrease in the number of subjects experiencing moderate anxiety/depression, compensated by an increase in those reporting slight anxiety/depression. However, our study did not replicate findings from a recent study indicating that individuals requiring frequent intracardiac electrical shocks post-ICD implantation had a poorer quality of life [12]. Other studies have also reported either minimal change or relatively improved quality of life in ICD-implanted patients compared to those receiving optimal medical treatment alone [13,14].

Our study yielded interesting findings regarding the assessment of quality of life in subjects who underwent cardiac resynchronization device implantation. We observed a positive impact of this cardiac device on quality of life, particularly evident from the third visit onward across the mobility, self-care, and usual activity modules. This positive impact was observed both in the early period (30-45 days after device implantation) and later periods (one and three years post-implantation). We attribute this effect to the restoration of heart chamber synchronization, leading to improved systolic function and reduced heart failure severity. These results prompt further consideration of cardiac device implantation in patients meeting modern recommendations and appropriate selection of target groups for implantation. Notably, intriguing data were obtained from the point of pain/discomfort module. Patients who underwent cardiac resynchronization therapy device implantation experienced an increase in moderate intensity pain/discomfort scores at the second and third visits, which returned to baseline levels in subsequent visits. This is likely attributed to the particularities of subjects' adaptation to a relatively large wound and foreign body, along with possible periodic diaphragmatic stimulation. These factors may also be linked to data obtained from the anxiety/depression module, where a notable increase in anxiety/depression rates was observed during the same period. The subsequent decrease in these rates from the fourth visit may be related to the restoration of synchronization of the heart's right and left sides, improvement in systolic function, and reduction in heart failure events. This factor is directly associated with the enhancement of subjects' quality of life. Notably, the improvement in anxiety/depression persisted throughout the study, affirming the positive impact of the cardiac resynchronization therapy device on the quality of life of relevant subjects. Our study's findings align with those of a 2013 publication, which reported a positive effect on the quality of life of patients in terms of the mental component nine months after cardiac resynchronization device implantation [15]. Furthermore, our study's results corroborate data from previous studies regarding the favorable impact of CRT on quality of life compared to ICD-implanted patients [16].

A limitation of our study may be the fact that the results of the study are based on the perception and evaluation of the subjective feelings of the patients.

## Conclusions

Pacemaker implantation in individuals with sick sinus syndrome and high-grade AV block demonstrated significant and highly reliable positive effects on quality of life in terms of mobility, self-care, usual activity, and anxiety/depression, both in the early and late post-pacemaker implantation periods. However, pacemaker implantation did not significantly affect the pain/discomfort module.

On the other hand, cardioverter-defibrillator implantation did not have a significant impact on quality of life modules, such as mobility, self-care, and usual activity, both in early and late periods. It did, however, somewhat improve the data of subjects with mild pain/discomfort and moderate anxiety/depression. Cardiac resynchronization therapy device implantation revealed a significant and highly reliable positive effect on quality of life in terms of mobility, self-care, and usual activity, both early and late after implantation. However, implantation of this type of cardiac device did not have a positive effect on the quality of life in the pain/discomfort module. An increase in the incidence of moderate-intensity pain/discomfort was observed in the early periods after implantation. As for the anxiety/depression module, a somewhat positive effect was observed in this direction on the quality of life in different periods after implantation.
